# Comparing distributed knowledge: the effects of visualization format and comparison strategy on task performance

**DOI:** 10.3389/fpsyg.2026.1684634

**Published:** 2026-02-10

**Authors:** Nicole Hynek, Dietrich Albert

**Affiliations:** Institute of Psychology, University of Graz, Graz, Austria

**Keywords:** cognitive load theory, comparative knowledge visualization, concept map, group knowledge integration, juxtaposition, proposition list, superimposition, visual comparison strategy

## Abstract

**Introduction:**

Teams can benefit from collaboration tools that make distributed knowledge more accessible and comparable when sharing and integrating information. Comparative knowledge visualizations serve this function by presenting multiple knowledge profiles within a shared display. This allows users to distinguish shared information from unshared information. Despite the widespread use of knowledge visualizations in collaborative settings, comparatively little is known about how specific design choices support comparing multiple knowledge profiles. This study examined how comparative knowledge visualizations support users’ understanding of distributed knowledge. The study focused on two core design decisions: how to represent conceptual knowledge and how to arrange multiple profiles within a shared display.

**Methods:**

We manipulated two core design decisions in comparative knowledge visualizations, each of which was implemented in two variants: knowledge representation format (concept maps versus proposition lists) and visual comparison strategy (juxtaposition versus superimposition). We also varied task complexity to test whether design advantages increase as comparison demands rise. In a 2 × 2 × 3 mixed-design experiment (*N* = 133), participants completed a visual comparison task in which they judged whether statements about the distribution of knowledge across three fictional group members were true or false based on the visualization. We assessed accuracy, response time, and perceived cognitive usability.

**Results:**

Comparison strategy showed a robust effect: superimposition yielded faster responses overall and higher accuracy under medium and high complexity. Knowledge format did not affect performance. Usability ratings indicated complementary advantages: superimposition was perceived as more helpful for comparing profiles and accessing group-level knowledge, whereas juxtaposition was rated clearer and more supportive for identifying individual knowledge.

**Discussion:**

The effectiveness of comparative knowledge visualizations depends on how multiple profiles are perceptually aligned and separated to match the epistemic goal (integrative comparison vs. source-specific inspection) and the processing demands of the task. The results provide evidence-based guidance for designing comparative displays that support identification of shared and unshared knowledge in collaborative work.

## 1 Introduction

Interdisciplinary collaboration is widely regarded as a promising approach for tackling knowledge-intensive tasks and complex problems. By combining diverse disciplinary perspectives, these collaborations enable richer problem representations that integrate different assumptions, viewpoints, and explanatory mental models. Such epistemic diversity can lead to more effective solutions than those developed by individuals or homogeneous groups (Blomkamp, 2018; Dillenbourg and Bétrancourt, 2006; Larson and Christensen, 1993; Maciver et al., 2021).

However, pooling and coordinating distributed knowledge from multiple contributors is cognitively demanding, especially when group members must consider several knowledge sources simultaneously. Under these conditions, the resulting information load can exceed working memory capacity. Group members may then overvalue familiar, overlapping content while neglecting contributions that are not shared (Dillenbourg and Bétrancourt, 2006; Stasser and Abele, 2020). This tendency is known as the shared-information or evaluation bias and is a well-documented barrier to effective group decision-making and problem-solving (Schulz-Hardt et al., 2000; Stasser and Titus, 1985, 2003; Stewart and Stasser, 1998; Stasser and Abele, 2020; Bang and Frith, 2017). Thus, successful knowledge integration requires group members to recognize relationships between their contributions and distinguish shared from unshared knowledge (Dehler Zufferey et al., 2010; Engelmann and Hesse, 2010, 2011). Consequently, groups may fail to leverage their expertise fully as they converge on decisions that reflect a disproportionate focus on a shared subset of information rather than on the full distribution of knowledge.

Externalizing knowledge through visual or tangible artifacts is a promising approach to help collaborators share and integrate their knowledge (Engelmann and Hesse, 2010, 2011; Oppl and Stary, 2019). Theories of distributed and external cognition (Hollan et al., 2000; Scaife and Rogers, 1996; Zhang and Norman, 1994) suggest that offloading reasoning from internal memory onto external resources (e.g., technology or knowledge artifacts) can reduce memory and coordination demands by altering a task’s information-processing requirements (Patterson et al., 2014; Risko and Gilbert, 2016; Heersmink, 2021; Skulmowski, 2023; Gilbert et al., 2023). In collaborative settings, external aids and actions, including digital tools and physical objects, can therefore support the team’s coordination and ease integration processes when cognitive resources are limited (Fiore and Wiltshire, 2016). Accordingly, such artifacts can function as scaffolds that structure information processing and regulate working memory demands (van Nooijen et al., 2024). They can also provide shared reference points (i.e., boundary objects) that make knowledge visible, support joint attention, and keep content open to inspection and negotiation (Arias and Fischer, 2000; Tergan and Keller, 2005; Engelmann et al., 2009; Engelmann and Hesse, 2010, 2011; Erkens and Bodemer, 2017, 2019; Oppl and Stary, 2019).

At the same time, externalization does not eliminate the core difficulty of integrating multiple contributors’ knowledge. Processing multiple knowledge sources remains cognitively demanding because users must align information across sources while maintaining intermediate results during verification, especially under constrained working memory (Cowan, 2010; Dillenbourg and Bétrancourt, 2006). Therefore, knowledge visualizations must be deliberately designed to meet the cognitive requirements of specific epistemic tasks (e.g., comparing knowledge within a group) and to support effective processing (Card et al., 1999; Albert and Steiner, 2005a, 2005b; Ghoniem et al., 2005). Meyer’s (2010) typology of knowledge visualizations specifies how design decisions relate to cognitive and epistemic functions across four interdependent dimensions: knowledge type (e.g., declarative vs. procedural), epistemic function (e.g., insight generation vs. group coordination), target audience (e.g., individual vs. group), and presentation format (e.g., sketch vs. map). Complementing this view, Tversky’s (2005) principles of visual cognition emphasize that visual structures should align with users’ mental organization of information (principle of congruence) and be easy and accurate to interpret (principle of apprehension).

### 1.1 Design dimensions of comparative knowledge visualization

Applied to collaborative settings, these frameworks imply that visualization design should be evaluated in terms of how well it represents group knowledge and supports intended epistemic operations, such as comparing knowledge across contributors to detect shared and unshared information. From this perspective, graph-based formats such as concept maps can represent knowledge in ways that facilitate visual comparison across multiple individuals. These formats often include conceptual and procedural elements and action sequences, and they are commonly used to represent semantically rich knowledge involved in reasoning and problem solving (Novak and Cañas, 2006; Keller and Tergan, 2005; Steiner et al., 2007; Meyer, 2010). For instance, concept maps depict conceptual knowledge as propositions, which are statements that connect two concepts (nodes) through a labeled relationship (Novak, 2004; Novak and Gowin, 1984). This spatial graph arrangement makes concepts and their semantic relations perceptually explicit, supporting efficient retrieval and relational reasoning (Novak and Cañas, 2006; Keller and Tergan, 2005). Visualization tools such as Mental Modeler (Gray et al., 2013) and M-Tool (van den Broek et al., 2021), as well as approaches such as the Knowledge and Information Awareness (KIA) approach (Engelmann et al., 2009; Engelmann and Hesse, 2010) implemented with CmapTools (Cañas et al., 2004; Cañas, 2005), have been developed as visual–spatial knowledge environments for sharing and integrating knowledge. Using such graph-based environments has been shown to enhance communication and support the integration of distributed knowledge in collaborative problem-solving tasks (Engelmann et al., 2009; Engelmann and Hesse, 2010, 2011; Engelmann et al., 2014; Keller et al., 2006).

Despite their benefits, comparatively little is known about how graph-based knowledge visualizations support epistemic operations in groups. In particular, the role of these visualizations in facilitating the visual comparison of information among group members lacks empirical foundation. However, this process is central to identifying shared and unshared knowledge (Pagendarm and Post, 1995; Engelmann et al., 2014). Importantly, the design of effective visual comparisons may depend not only on how knowledge is represented within individual profiles, but also on how multiple profiles are arranged for comparison within a shared display. Thus, comparative knowledge visualizations can be understood as epistemic interfaces that combine a representation format for externalizing individual conceptual knowledge and a comparison strategy for aligning multiple profiles.

**Figure 1:**
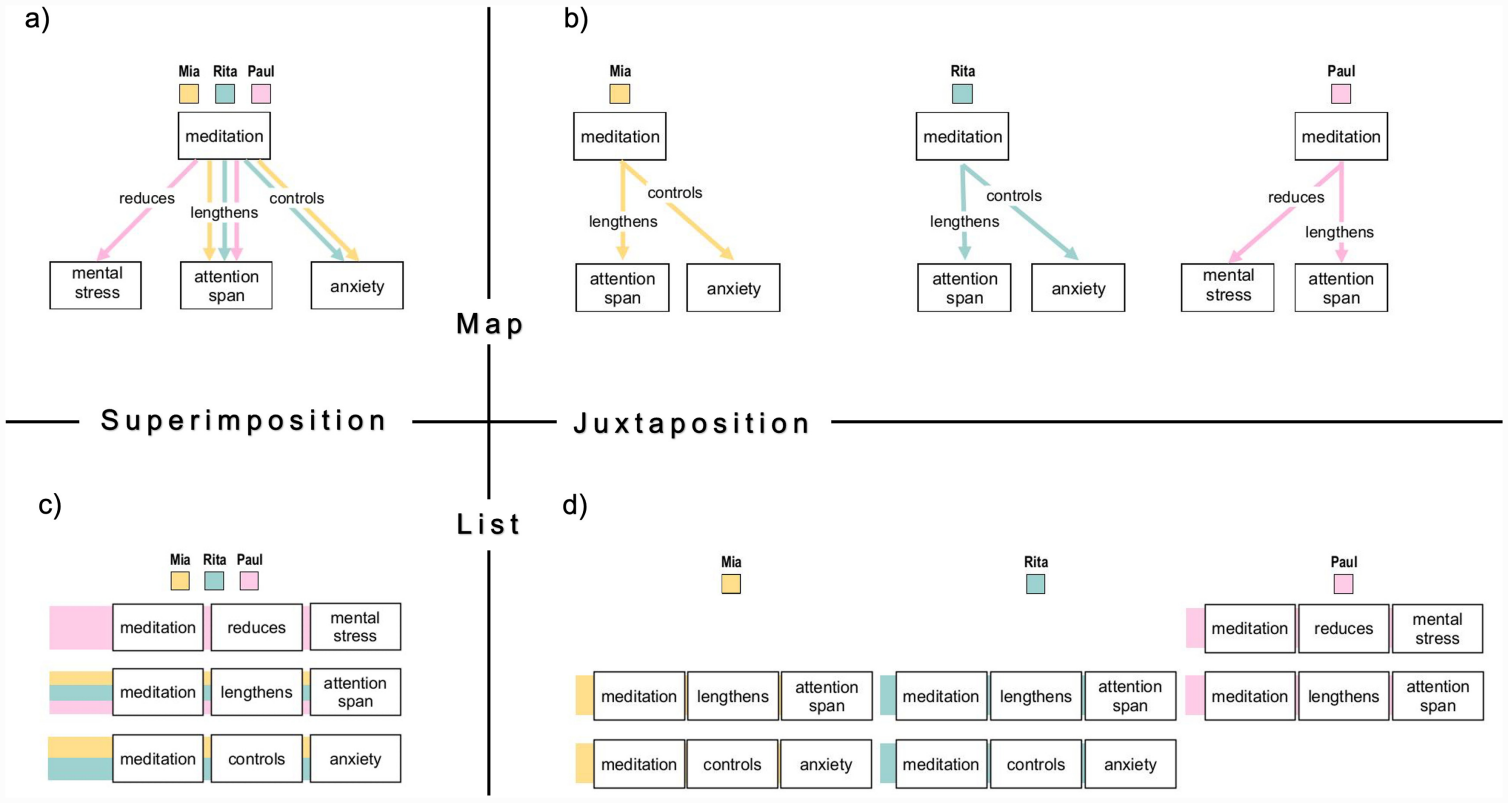
Simplified example of a full 2 × 2 visualization set displaying three knowledge propositions. The set comprises all combinations of knowledge format (concept maps vs. proposition lists) and comparison strategy (juxtaposition vs. superimposition): **(a)** Superimposed concept map, **(b)** Juxtaposed concept maps, **(c)** Superimposed proposition list, and **(d)** Juxtaposed proposition lists. To ensure structural comparability, proposition lists mirror the hierarchical layout of the corresponding concept maps. In both formats, each concept is enclosed in a rectangular box. Semantic relations are represented as boxed statements in proposition lists, whereas in concept maps, labeled links are placed above connecting lines to enhance readability. A consistent color-coding scheme is used to differentiate the knowledge contributions of the three fictional individuals: orange = person A (Mia), turquoise = person B (Rita), pink = person C (Paul).

To examine this interface function, we implemented a 2 × 2 comparative visualization design that systematically combined two knowledge representation formats (concept maps vs. proposition lists) and two comparison strategies (superimposition vs. juxtaposition), as shown in Figure 1. We tested how these design dimensions shape users’ understanding of distributed knowledge by measuring participants’ performance in a propositional verification task (see Section 2.2.2).

The first design dimension addressed the format of knowledge representation and compared concept maps, a visual–spatial graph format, with proposition lists, a linear-sequential list format. Both formats were designed to make semantic relations explicit and accessible within individual knowledge profiles. Concept maps depict concepts as nodes connected by labeled links (see Figures 1a,b) and can reduce redundancy by reusing concepts across propositions. From a cognitive perspective, it is assumed that the node–link structure can transfer relational reasoning from working memory to the perceptual system (Larkin and Simon, 1987; Sweller et al., 1998; Tversky et al., 2007). Proposition lists present each proposition as a sentence in a line-by-line format (see Figures 1c,d). This format may require more sequential search and impose higher extraneous processing demands in comparison tasks (Maslianko and Sielskyi, 2021). However, proposition lists may support item-level verification by presenting propositions in an explicitly segmented verbal structure, which could reduce spatial search demands when users must check specific statements.

The second design dimension addressed the arrangement of multiple knowledge profiles for comparison and contrasted superimposition with juxtaposition (Gleicher et al., 2011). Superimposed views overlay profiles in a single frame, enabling direct visual comparison within the “eye span” (Tufte and Graves-Morris, 1983) and facilitating the perception of overlaps and differences (Cleveland and McGill, 1984; Windhager et al., 2020). In contrast, juxtaposed views display individual profiles side by side (see Figures 1b,d). While this preserves source separation, it increases the need for attentional shifts and working memory when users must align corresponding information across displays (Gleicher et al., 2011; Meulemans et al., 2016; Wolfe, 2020; Matlen et al., 2020).

### 1.2 Task complexity as a boundary condition for visualization effectiveness

Furthermore, design advantages of comparative visualizations should become most apparent as comparison demands increase. From a cognitive load perspective, increasing task complexity primarily raises intrinsic load because more informational elements and relations must be processed and coordinated simultaneously (Sweller et al., 2019; Fiore et al., 2017). Under such conditions, visualization design should matter most when it reduces extraneous processing (e.g., attentional switching, mental alignment, and spatial search) and supports task-relevant processing by making relevant structure easier to perceive and use (Sweller et al., 2019; Tversky, 2005). Task complexity can therefore be manipulated (e.g., by varying how many propositions and sources must be coordinated) to increase comparison demands and serve as a boundary condition for when design advantages are most likely to emerge.

Against this backdrop, we examined the comparative function of visualization design, guided by the principle of application validity. This principle is defined as the extent to which a design supports the successful completion of an intended epistemic task (Ware et al., 2002; Steiner and Albert, 2017a; Steiner and Albert, 2017b). Accordingly, we employed a task-based evaluation approach that assessed visualization effectiveness under systematically varied complexity levels of the propositional verification task. In addition to these objective indicators, we examined users’ perceived cognitive usability, namely how well each design supports key epistemic operations, such as comparing knowledge and accessing group- versus individual-level information (see Section 2.4.3).

The present study addressed the following research questions: (RQ1) How do knowledge representation format (concept maps vs. proposition lists) and visual comparison strategy (superimposition vs. juxtaposition) affect performance in identifying shared and unshared knowledge, as reflected in response time and accuracy? (RQ2) Do these format- and strategy-related performance effects depend on task complexity (low, medium, high), such that differences become more pronounced as comparison demands increase? (RQ3) How do participants rate the cognitive usability of the visualizations for key epistemic operations as a function of knowledge representation format and comparison strategy?

Based on prior literature and the implied design advantages, we derived the following hypotheses. We predicted that concept maps would result in faster response times and greater accuracy than proposition lists (H1a) and that this advantage would increase with task complexity (H1b). We also predicted that superimposed views would outperform juxtaposed views (H2a), with the advantage becoming more pronounced with greater complexity (H2b). Finally, we hypothesized that combining concept maps and superimposed views would produce the best outcomes (H3a), with this advantage increasing with task complexity (H3b). The cognitive usability ratings to evaluate the subjective perception of the visual design were analyzed in an exploratory manner.

## 2 Methods

### 2.1 Participants

The final sample consisted of 133 participants, drawn from an initial pool of 137. Four participants were excluded from all analyses due to incomplete data on most rating scales, implausibly short response times (i.e., <6 s) across trials, and error rates exceeding 80%, suggesting insufficient task engagement. Participants were randomly assigned to the concept map condition (*n* = 63; 21 men, 39 women, 3 nonbinary; age: *M* = 36.2, *SD* = 13.3) or the proposition list condition (*n* = 70; 27 men, 38 women, 5 nonbinary; age: *M* = 35.5, *SD* = 11.6). The sample spanned a broad age range (18–65) and was relatively well educated, with comparable educational attainment across conditions (concept map: 70% university degree, 25% high school, 5% compulsory schooling; proposition list: 75% university degree, 23% high school, 2% compulsory schooling). Participants were recruited via social media platforms and university email lists. Eligibility criteria were normal color vision and German language proficiency of at least C1; individuals not meeting these criteria were asked not to participate. No monetary compensation or course credit was provided.

### 2.2 Material and task

#### 2.2.1 Stimuli: comparative knowledge visualizations

A total of nine visualization sets were created using yEd (yWorks^1^). Each set contained four visualizations representing all possible combinations of two knowledge formats (concept maps vs. proposition lists) and two comparison strategies (juxtaposition vs. superimposition). Across the three fictional individuals shown in each visualization, the displayed knowledge comprised a total of 16 propositions on a mental health topic, with some propositions shared and others unique to individual profiles. Propositions were formulated as declarative statements linking two concepts via a semantic relation (e.g., meditation reduces mental stress), using plain, non-technical language throughout. All visualizations were presented as static images with a fixed resolution of 1,280 × 1,024 pixels and had no interactive features. Individual profiles were distinguished using a consistent color-coding scheme: orange (#ffdb81) for person A, turquoise (#9fd1cf) for person B, and pink (#ffb7de) for person C (see Figure 1).

#### 2.2.2 Visual comparison task: verifying statements across three profiles

Participants completed an 18-trial visual comparison task to evaluate the effectiveness of the visualization design in facilitating quick and accurate identification of shared and unshared knowledge. In each trial, participants decided whether a written statement was true or false based on the information shown in the visualization. Each statement described how specific knowledge elements (propositions) were distributed among three fictional group members. Participants compared the depicted knowledge profiles to verify each statement (e.g., determining whether a proposition was shared by multiple members or unique to one).

The complexity of the task was manipulated by varying the number of propositions and individuals referenced in the statement. This yielded three predefined levels: low, medium, and high. In line with cognitive load theory, task complexity is defined as the number of informational elements in a statement that must be processed and integrated to complete the task. Task complexity differs from task difficulty, which refers to the subjective mental effort experienced while performing the task (Huang et al., 2009; Liu and Li, 2012; Newton et al., 2023). Low complexity involved one proposition and one individual (e.g., “*Paul knows that meditation reduces mental stress*”). Medium complexity involved one proposition distributed across three individuals (e.g., “*Mia and Rita, but not Paul, know that meditation controls anxiety*”). High complexity involved two propositions distributed across three individuals (e.g., “*All three know that meditation lengthens attention span, but only Rita knows that physical fitness reduces the risk of certain diseases*”). Consequently, higher-complexity statements require the coordination of more elements and therefore impose higher intrinsic processing demands than low-complexity statements, regardless of the visualization design. This manipulation allowed us to test whether visualization strategies support statement verification under increasing processing demands (i.e., higher intrinsic task complexity).

### 2.3 Procedure

The study followed a 2 × 2 × 3 mixed factorial design with knowledge format (concept map vs. proposition list) as a between-subjects factor and comparison strategy (juxtaposition vs. superimposition) and task complexity (low, medium, high) as within-subjects factors. Participants were assigned to one knowledge format condition (concept map or proposition list) and completed the visual comparison task under both comparison strategies. Comparison strategy was manipulated within participants in two blocked phases (nine trials each), whereas task complexity varied within each block. To control for sequence effects, the two comparison strategies were administered in two counterbalanced block orders (juxtaposition → superimposition vs. superimposition → juxtaposition), yielding four between-subject conditions (knowledge format × block order) to which participants were randomly assigned.

After providing informed consent, participants completed demographic questions and the familiarity items described in Section 2.4.1. Participants then completed the visual comparison task in two blocks of nine trials. At the beginning of each block, participants were shown a simplified example of the upcoming visualization display and completed two practice trials of moderate complexity with explanatory feedback. The first block used one comparison strategy (juxtaposition or superimposition), and the second block used the alternative strategy. Within each block, comparison strategy was held constant, and participants completed three trials per complexity level (low, medium, high), presented in randomized order. Trial materials were drawn from a larger pool of preconstructed statements and corresponding visualizations, such that participants did not necessarily see identical item sets. After each block, participants rated the cognitive usability of the provided group knowledge visualization using four items (see Section 2.4.3).

### 2.4 Measures

#### 2.4.1 Participant information

Participants completed a short questionnaire assessing their demographic background and familiarity with different visual information formats. Demographic information included gender (male, female, or other), age (in years), and highest level of completed education (primary, secondary, high school, or college/university).

To assess participants’ familiarity with information visualizations comparable to those used in the study, we asked them to rate their agreement with three items on a 6-point Likert scale (0 = *no experience*, 5 = *high experience*); familiarity ratings did not differ between the concept map and proposition list groups (all *ps* ≥ 0.38; see Supplementary material S1). The items were:

- Data visualization: “*I have experience with statistical data visualizations (*e.g.*, bar charts, scatterplots).*”
- Network visualization: “*I have experience with network visualizations (*e.g.*, node-link diagrams).*”
- Table visualization: “I have experience with table visualizations (e.g., spreadsheet-like matrices).”

#### 2.4.2 Task performance measures

The experiment was implemented as a web-based application using the jsPsych framework^2^. Participants responded via mouse or trackpad, and accuracy and response times were recorded automatically. Accuracy rates (ARs) captured response correctness in the visual comparison task. Each trial was scored as 1 for a correct true/false decision and 0 for an incorrect decision. Within each task block, accuracy was summarized separately for each task complexity level as the number of correct responses across the three trials (range = 0–3). Response times (RTs) captured processing efficiency and were defined as the latency (in milliseconds) between trial onset and the participant’s response.

#### 2.4.3 Cognitive usability ratings

After each task block, participants answered a short, four-item questionnaire assessing the perceived cognitive usability of the provided group visualization. The items addressed conceptually distinct functions and were formulated to ensure high face validity. Participants rated each item on a six-point Likert scale ranging from 0 (not at all) to 5 (completely). The items targeted the following functional dimensions:

- Visual comparison: “*The visualization made it easy to compare multiple knowledge profiles.*”
- Visual clarity: “*The visualization was clear and easy to read.*”
- Group knowledge access: “*It was easy to see what the group collectively knew.*”
- Individual knowledge access: “*It was easy to identify what each group member knew.*”

## 3 Results

Prior to statistical analysis, the data were screened for potential outliers. Isolated extreme values in response time data were identified for 11 participants (>80 s), suggesting temporary task disengagement. To balance sensitivity and data retention, we applied Tukey’s interquartile range (IQR) method using a liberal cutoff multiplier of 2.2 (Tukey, 1977; cf. Wilcox, 2012). Single-trial outliers were winsorized–that is, replaced with the nearest non-outlier value within the same distribution (Erceg-Hurn and Mirosevich, 2008).

All analyses were preceded by assumption checks for normality, homogeneity of variance, and sphericity. Where applicable, Greenhouse–Geisser corrections were applied when sphericity was violated, and Bonferroni-adjusted *post hoc* tests followed significant omnibus effects.

### 3.1 Task performance

We analyzed task performance using a mixed-design ANOVA with knowledge format (concept maps vs. proposition lists) as a between-subjects factor and comparison strategy (superimposition vs. juxtaposition) and task complexity (low, medium, high) as within-subjects factors. The model included two dependent measures, response time (RT; correct trials only) and accuracy rate (AR; 0–3 correct per complexity level within each task block), which are reported separately.

#### 3.1.1 Response time

The response time increased with task complexity (Table 1). The mixed ANOVA showed a robust main effect of task complexity, *F*(1.83, 239.72) = 162.52, *p* < 0.001, *ηp*^2^ = 0.554. Collapsed across knowledge format and comparison strategy, estimated marginal means (EMMs) increased from *M* = 20.58 s (*SE* = 0.69) at low complexity to *M* = 23.66 s (*SE* = 0.86) at medium complexity and *M* = 33.10 s (*SE* = 1.03) at high complexity (see also Table 2). Bonferroni-adjusted post hoc comparisons indicated that all three complexity levels differed (all *ps* < 0.001), confirming that the complexity manipulation increased processing demands.

**Table 1:** Estimated marginal means for response time (s) by knowledge format (map vs. list), comparison strategy (superimposition vs. juxtaposition), and task complexity (low, medium, high).

	Concept map								Proposition list							
	Superimposition				Juxtaposition				Superimposition				Juxtaposition			
	*EMM*	*SE*	*95% CI*		*EMM*	*SE*	*95% CI*		*EMM*	*SE*	*95% CI*		*EMM*	*SE*	*95% CI*	
			*LL*	*UL*			*LL*	*UL*			*LL*	*UL*			*LL*	*UL*
Task complexity																
Low	19.45	1.31	16.86	22.04	22.68	1.24	20.23	25.12	18.46	1.24	16.00	20.91	21.76	1.17	19.44	24.08
Medium	23.12	1.12	20.93	25.32	27.72	1.66	24.43	31.01	18.35	1.05	16.27	20.43	25.43	1.58	22.31	28.56
High	31.84	1.67	28.54	35.14	37.84	2.06	33.76	41.92	27.92	1.58	24.79	31.05	34.80	1.95	30.93	38.66

Values are estimated marginal means (EMMs) from the mixed-design ANOVA. Response times are in seconds and based on correct trials only. SE = standard error. CI = 95% confidence interval (LL = lower limit; UL = upper limit).

**Table 2:** Estimated marginal means for response time (s), collapsed across the other factors.

Factor	EMM	SE	95% CI	
			*LL*	*UL*
Knowledge format				
Concept maps	27.11	1.10	24.92	29.30
Proposition lists	24.29	1.05	22.37	26.53
Comparison strategy				
Superimposition	23.19	0.78	21.66	24.72
Juxtaposition	28.37	0.98	26.42	30.32
Task complexity				
Low	20.58	0.69	19.21	21.96
Medium	23.66	0.86	21.97	25.35
High	33.10	1.03	31.06	35.14

Values are estimated marginal means (EMMs) from the mixed-design ANOVA. Response times are in seconds and based on correct trials only. SE = standard error. CI = 95% confidence interval (LL = lower limit; UL = upper limit).

Comparison strategy also affected processing efficiency in terms of RT (see Table 3). Participants responded faster with superimposed than with juxtaposed views, *F*(1, 131) = 33.16, *p* < 0.001, *ηp*^2^ = 0.202; collapsed EMMs were *M* = 23.19 s (*SE* = 0.78) for superimposition and *M* = 28.37 s (*SE* = 0.98) for juxtaposition (Table 2). However, the strategy × complexity interaction did not reach significance. Bonferroni-adjusted simple-effects comparisons nevertheless showed faster responses under superimposition at each complexity level (all *ps* ≤ 0.003), with numerically larger differences at higher complexity (see Table 1).

**Table 3:** Mixed ANOVA results for response time (s).

Effect	df1	df2	*F*	*p*	ηp^2^
Knowledge format	1	131	3.02	0.084	0.023
Comparison strategy	1	131	33.16	< 0.001	0.202
Task complexity	1.83	239.72	162.52	< 0.001	0.554
Format × Complexity	1.83	239.72	2.07	0.133	0.016
Format × Strategy	1	131	0.41	0.525	0.003
Strategy × Complexity	1.89	247.73	2.79	0.067	0.021
Format × Strategy × Complexity	1.89	247.73	0.37	0.678	0.003

Greenhouse–Geisser correction was applied for effects involving task complexity where Mauchly’s test indicated sphericity violations. Bonferroni-adjusted post hoc tests were used following significant omnibus effects.

The knowledge format did not reveal any significant differences in response times, nor any interaction effects related to strategy or complexity (all *ps* ≥ 0.084; see Table 3). Overall, RT results supported the predicted benefit of superimposition (H2a) but did not provide clear evidence for an increased advantage under higher task complexity (H2b), nor for format-related advantages (H1a/H1b) or the predicted format × strategy benefit (H3a/H3b).

#### 3.1.2 Accuracy rate

The accuracy rate decreased with task complexity (see Table 4). The mixed ANOVA revealed a significant main effect of task complexity, *F*(2, 262) = 10.69, *p* < 0.001, *ηp*^2^ = 0.075. Collapsed across knowledge format and comparison strategy, EMMs (0–3 correct per complexity level) declined from *M* = 2.870 (*SE* = 0.024) at low complexity to *M* = 2.831 (*SE* = 0.027) at medium complexity and *M* = 2.726 (*SE* = 0.033) at high complexity (see Table 5). Bonferroni-adjusted comparisons indicated no difference between low and medium complexity (*p* = 0.595), but significant declines from low to high (*p* < 0.001) and from medium to high (*p* = 0.007).

**Table 4:** Estimated marginal means for accuracy rate (0–3) by knowledge format (map vs. list), comparison strategy (superimposition vs. juxtaposition), and task complexity (low, medium, high).

	Concept map										Proposition list									
	Superimposition					Juxtaposition					Superimposition					Juxtaposition				
	*EMM*	*SE*	*95% CI*		*% corr*	*EMM*	*SE*	*95% CI*		*% corr*	*EMM*	*SE*	*95% CI*		*% corr*	*EMM*	*SE*	*95% CI*		*% corr*
			*LL*	*UL*				*LL*	*UL*				*LL*	*UL*				*LL*	*UL*	
Task complexity																				
Low	2.81	0.05	2.71	2.91	94	2.86	0.05	2.76	2.96	95	2.93	0.05	2.83	3.02	98	2.89	0.05	2.79	2.98	96
Medium	2.87	0.04	2.79	2.95	96	2.81	0.06	2.68	2.94	94	2.93	0.04	2.85	3.01	98	2.71	0.06	2.59	2.83	90
High	2.79	0.05	2.69	2.89	93	2.52	0.08	2.36	2.69	84	2.90	0.05	2.80	2.99	97	2.69	0.08	2.53	2.84	89

Accuracy values are estimated marginal means (EMMs) from the mixed-design ANOVA and reflect the mean number of correct responses per task complexity level (range 0–3; three trials per level). Percent correct (% corr) was computed as (EMM/3) × 100, rounded to whole percentages. SE = standard error. CI = 95% confidence interval (LL = lower limit; UL = upper limit).

**Table 5:** Estimated marginal means for accuracy rate (0–3), collapsed across the other factors.

Factor	EMM	SE	95% CI		% corr
			LL	UL	
Knowledge format					
Concept maps	2.78	0.03	2.72	2.84	93
Proposition lists	2.84	0.02	2.78	2.90	95
Comparison strategy					
Superimposition	2.87	0.02	2.82	2.91	96
Juxtaposition	2.75	0.03	2.69	2.81	92
Task complexity					
Low	2.87	0.02	2.82	2.92	96
Medium	2.83	0.03	2.78	2.88	94
High	2.73	0.03	2.66	2.79	91

Accuracy values are EMMs from the mixed ANOVA model and refer to the mean number of correct responses per complexity level (range 0–3; three trials per level). Percent correct (% corr) was computed as (EMM/3) × 100 and rounded to whole percentages. SE = standard error. CI = confidence interval; LL/UL = lower/upper limit.

Comparison strategy also affected the accuracy with which participants completed the task (see Table 6). Participants were more accurate with superimposition than juxtaposition (*F*(1, 131) = 16.14, *p* < 0.001, *ηp*^2^ = 0.110). The collapsed EMMs were *M* = 2.872 (*SE* = 0.022) for superimposition and *M* = 2.746 (*SE* = 0.031) for juxtaposition (see also Table 5). This benefit depended on task complexity, as indicated by a significant strategy × complexity interaction, *F*(1.87, 245.41) = 4.76, *p* = 0.011, *ηp*^2^ = 0.035. Simple-effects comparisons showed that the superimposition advantage was not reliable at low complexity (*p* = 0.961) but was significant at medium (*p* = 0.008) and high complexity (*p* < 0.001), with the largest advantage in the high-complexity condition.

**Table 6:** Mixed ANOVA results for accuracy rate (0–3; three trials per complexity level).

Effect	df1	df2	*F*	*p*	ηp^2^
Knowledge format	1	131	2.17	0.143	0.016
Comparison strategy	1	131	16.14	< 0.001	0.110
Task complexity	2	262	10.69	< 0.001	0.075
Format × Complexity	2	262	2.88	0.058	0.022
Format × Strategy	1	131	0.97	0.326	0.007
Strategy × Complexity	1.87	245.41	4.76	0.011	0.035
Format × Strategy × Complexity	1.87	245.41	0.89	0.407	0.007

Greenhouse–Geisser correction was applied only for effects involving the strategy × complexity, for which Mauchly’s test indicated a sphericity violation. Bonferroni-adjusted post hoc tests were used following significant omnibus effects.

No effects involving the knowledge format were significant (Table 6), although the format × complexity interaction was marginal (*p* = 0.058). In sum, accuracy results supported the predicted advantage of superimposition (H2a) and its amplification under higher task complexity (H2b) but did not support format advantages (H1a/H1b) or the predicted format × strategy benefit (H3a/H3b).

### 3.2 Cognitive usability ratings

To assess perceived cognitive usability, we analyzed four ratings collected after each task block (visual comparison, visual clarity, access to group knowledge, and access to individual knowledge). For each rating, we conducted a 2 (representation format; between-subjects) × 2 (comparison strategy; within-subjects) mixed ANOVA. Descriptive statistics are reported in Table 7, and inferential statistics are summarized in Table 2. Because multiple usability tests were conducted on related outcomes, Bonferroni/Holm adjustments were applied; all effects reported as significant remained significant after correction (corrected *p* < 0.007).

**Table 7:** Means and standard deviations for each usability dimension by visualization format (concept map vs. proposition list) and comparison strategy (juxtaposed vs. superimposed).

Usability dimension	Concept map		Proposition list	
	Superimposition	Juxtaposition	Superimposition	Juxtaposition
Group Knowledge	3.41 (*1.33*)	2.33 (*1.49*)	2.93 (*1.34*)	2.74 (*1.61*)
Individual Knowledge	3.06 (*1.47*)	3.48 (*1.29*)	2.88 (*1.43*)	3.43 (*1.46*)
Knowledge Comparison	3.54 (*1.27*)	3.24 (*1.21*)	3.32 (*1.19*)	2.81 (*1.34*)
Visual Clarity	2.49 (*1.31*)	2.98 (*1.21*)	2.36 (*1.19*)	2.63 (*1.27*)

Ratings were given on a 6-point Likert scale from 0 (not at all) to 5 (completely); higher values indicate greater perceived cognitive usability. Italicized values indicate standard deviations (SD).

Visual comparison. Superimposed views were rated as more helpful for comparing distributed knowledge than juxtaposed views, reflected in a significant main effect of comparison strategy, *F*(1, 130) = 10.28, *p* = 0.002, *ηp*^2^ = 0.073. However, no main effect was found for knowledge format, nor was there a knowledge format × comparison strategy interaction (see Table 8).

**Table 8:** Inferential statistics for cognitive usability ratings from the four separate mixed ANOVAs.

Usability dimension	Effect	df1	df2	*F*	*p*	ηp^2^
Visual comparison						
Representation format		1	130	3.27	0.073	0.025
Comparison strategy		1	130	10.28	0.002	0.073
Format × Strategy		1	130	0.66	0.417	0.005
Visual clarity						
Representation format		1	129	1.96	0.164	0.015
Comparison strategy		1	129	11.05	0.001	0.079
Format × Strategy		1	129	1.38	0.243	0.011
Group knowledge access						
Representation format		1	131	0.00	0.962	0.000
Comparison strategy		1	131	13.33	<0.001	0.092
Format × Strategy		1	131	6.66	0.011	0.048
Individual knowledge access						
Representation format		1	131	0.18	0.675	0.001
Comparison strategy		1	131	11.00	0.001	0.077
Format × Strategy		1	131	0.20	0.652	0.002

ηp^2^ values were computed from the reported F and degrees of freedom. Multiple-testing adjustments (Bonferroni/Holm) were applied as described in the text.

Visual clarity. Juxtaposed views were rated as clearer and more readable than superimposed views, reflected in a significant main effect of comparison strategy, *F*(1, 129) = 11.05, *p* = 0.001, *ηp*^2^ = 0.079. Once more, the main effect of knowledge format was not significant, nor was there a knowledge format × comparison strategy interaction (see Table 8).

Access to group knowledge. Ratings favored superimposition overall, *F*(1, 131) = 13.33, *p* < 0.001, ηp^2^ = 0.092, and this effect depended on representation format, as shown by a significant format × strategy interaction, *F*(1, 131) = 6.66, *p* = 0.011, *ηp*^2^ = 0.048 (Table 2). Follow-up comparisons indicated that superimposition increased perceived access to group knowledge in the concept map condition, *t*(62) = 4.12, *p* < 0.001, *d* = 0.52, but not in the proposition list condition, *t*(69) = 0.81, *p* = 0.42, *d* = 0.10.

Access to individual knowledge. Juxtaposed views were rated as more helpful for identifying individual knowledge than superimposed views, reflected in a significant main effect of comparison strategy, *F*(1, 131) = 11.00, *p* = 0.001, *ηp*^2^ = 0.077 (Table 2). A paired-samples t-test confirmed the comparison effect, *t*(132) = −3.35, *p* = 0.001, *d* = 0.29. However, neither the main effect of format nor the interaction effect was significant (see Table 8).

## 4 Discussion

This study examined how two fundamental design features of comparative group knowledge visualizations affect users’ ability to identify shared and unshared knowledge within a group. These features are the knowledge format (concept maps versus proposition lists) and the visual comparison strategy (juxtaposition versus superimposition). The results revealed a significant main effect for comparison strategy: Participants completed visual comparison tasks more accurately and efficiently with superimposed views than with juxtaposed ones. In contrast, no main effect was found for knowledge format, suggesting that concept maps and proposition lists were equally usable for the given task. Furthermore, performance differences between the comparison strategies increased with task complexity, indicating that perceptual alignment becomes more beneficial under higher cognitive load. These results will be discussed in more detail in the following sections.

### 4.1 Knowledge representation format: structural richness is not sufficient

We expected concept maps to outperform proposition lists because of their spatial organization of semantically interconnected information. These node-link structures are thought to promote semantic integration, pattern recognition, and conceptual understanding by grouping related ideas together, which facilitates perceptual grouping and semantic chunking (Novak and Cañas, 2008; Budé et al., 2009; Tergan, 2005; Engelmann and Hesse, 2010; Meyer, 2010). From a cognitive science perspective, concept maps are regarded as reducing relational inference load by offloading integration processes to the visual display, thereby supporting the construction of coherent mental models (Larkin and Simon, 1987; Johnson-Laird, 1983). However, concept maps can also impose additional demands on visual attention and spatial working memory, particularly when precise, item-level comparisons are required (Ware, 2004; Healey and Enns, 2012; Cowan, 2001).

Contrary to our hypothesis, participants performed equally well when using concept maps or proposition lists. This challenges the assumption that structurally richer graph-based representations provide superior cognitive support for tasks requiring the identification of shared and unshared conceptual knowledge across sources (Tergan and Keller, 2005; Engelmann and Hesse, 2010, 2011; Engelmann et al., 2014). Instead, the findings support the view that representational effectiveness depends on how well a format aligns with the cognitive and epistemic demands of the task (Ghoniem et al., 2005; Steiner and Albert, 2017a, 2017b).

In the present study, participants were tasked with identifying which of three individuals possessed a particular piece of knowledge or proposition. This involved verifying discrete conceptual units at the item level–a task that is potentially better supported by a linear, sentence-based list structure. The sequential layout of these lists may have facilitated visual search by reducing spatial disorientation and the demand on spatial working memory (Potelle and Rouet, 2003; van Oostendorp and Goldman, 1999).

These findings align with research in information visualization showing that linear, text-based representations can support efficient search and verification, particularly when tasks require stepwise access to specific content (Larkin and Simon, 1987). In our study, proposition lists presented clearly segmented sentences in a linear, top-down format, likely enabling users to conduct line-by-line comparisons externally without mentally tracking spatial relations or integrating content across a broader visual field (Maslianko and Sielskyi, 2021).

From this perspective, concept maps may offer advantages for tasks involving open-ended reasoning, structural insight, or conceptual exploration. However, in structured comparison tasks focused on factual verification, structural richness alone does not necessarily translate into greater comparative functionality. Future research could employ within-subject designs in which participants work with both concept maps and proposition lists across various task types. These studies would clarify whether concept maps provide greater support in tasks with higher semantic complexity or open-ended inferential demands and whether proposition lists are more effective for source-specific verification. Such studies would clarify how representational benefits depend on cognitive load and task requirements, providing a more informed basis for matching visualization formats to specific cognitive goals.

### 4.2 Visual comparison strategy: the role of perceptual alignment

The type of visual comparison strategy used had a robust and consistent influence on task performance. Participants responded faster and more accurately when using superimposed views than juxtaposed views to identify how knowledge was distributed among group members.

However, the extent to which this advantage depended on task complexity differed between response time and accuracy. Superimposition yielded faster processing at all complexity levels for response times, but the strategy × complexity interaction did not reach conventional significance. This indicates that the speed advantage was stable across levels of task demand. For accuracy, however, the benefit of the strategy increased with task complexity. Performance differences were negligible at low complexity but became reliable at medium and high complexity. The largest advantage was observed at high complexity. This pattern suggests that perceptual alignment through superimposition generally supports efficient comparisons and is particularly consequential for accuracy when tasks demand the maintenance and integration of multiple propositions during verification.

In line with previous research, these results support the notion that superimposed views offer cognitive advantages when users must detect structural correspondences across multiple sources, especially under high task complexity or when semantic proximity facilitates grouping (Gleicher et al., 2011; Javed et al., 2010; Windhager et al., 2020).

From a cognitive load perspective, this pattern shows that perceptual alignment decreases extraneous load by enabling users to compare related knowledge elements within a single, integrated view (Sweller et al., 2019; Keller and Grimm, 2005). This minimizes attentional shifts, mental alignment, and spatial memory operations, especially when users must detect overlapping propositions or conceptual gaps (Ware, 2004; Gleicher et al., 2011).

From a design perspective, these results support comparison strategies that actively promote perceptual integration, especially in tasks involving complex, distributed information. Juxtaposed views preserve source separability and facilitate content attribution or individual perspective tracking (Bodemer and Scholvien, 2008; Engelmann et al., 2009). However, superimposed views better support integrative reasoning by offloading structural alignment onto the visual display itself (Gleicher et al., 2011; Windhager et al., 2020).

The cognitive usability ratings of the participants further support these implications, as discussed in the following section.

### 4.3 Cognitive usability: functional differences of comparison strategies

To examine how participants experienced the cognitive affordances of the visualizations, we asked them to evaluate each group visualization format across four key dimensions of cognitive usability: visual clarity; ease of comparing knowledge; ease of accessing group-level knowledge; and ease of accessing individual knowledge. These ratings provide a subjective complement to objective performance data, capturing how participants perceived the alignment between the visual design and the epistemic demands of the task.

Overall, the results suggest that the two comparison strategies differ systematically in their epistemic affordances. Superimposed views were rated as more helpful for comparing distributed knowledge and gaining an overview of group-level content. By contrast, juxtaposed views were perceived as more effective for identifying individual contributions and were rated higher in visual clarity. This functional divergence aligns with prior information visualization findings: superimposition integrates multiple sources into a shared display. This reduces the need for internal coordination and enables direct perceptual alignment across representations (Gleicher et al., 2011; Gleicher, 2017; Matlen et al., 2020; Windhager et al., 2020). From a cognitive perspective, this alignment reduces the demands on working memory by offloading the processes of mental integration onto the visual display (Sweller et al., 1998; Cowan, 2010). This supports the efficient detection of overlaps and redundancies across knowledge profiles.

In contrast, juxtaposition spatially separates individual representations and preserves their source-specific structure. This layout is ideal for tasks in which users want to isolate and explore a single contributor’s knowledge without interference from others. By presenting each profile in a separate visual space, juxtaposed layouts reduce perceptual complexity and facilitate selective attention, which may be particularly beneficial when individual accountability or knowledge origin is relevant (Engelmann et al., 2009; Dehler Zufferey et al., 2010; Erkens and Bodemer, 2017, 2019).

Notably, the perceived advantage of superimposition for accessing group-level knowledge was only observed in the concept map condition. When participants worked with proposition lists, both comparison strategies received similar ratings. This suggests that the cognitive benefit of perceptual alignment is particularly relevant when semantic content is distributed across a graph-based, spatial layout. Concept maps represent conceptual propositions as node-link structures that arrange elements spatially across the canvas. Although juxtaposed maps use consistent node placement across individual profiles, superimposition enhances the integrative function by directly overlaying corresponding nodes and links into a single, shared frame. This reduces the need for attentional shifts and mental integration across multiple representations, supporting direct visual detection of shared elements through proximity-based perceptual grouping.

In contrast, proposition lists present knowledge sequentially in a sentence-based format, where conceptually related content is already grouped via verbal structure. Here, the added benefit of visual alignment–such as that enabled by superimposition–may offer less perceptual or cognitive advantage, as the linear format already guides users through semantically structured content without requiring spatial integration.

Taken together, these findings suggest that the perceived cognitive benefit of a certain type of comparative visualization does not follow a one-size-fits-all logic. Rather, it emerges from the functional alignment between layout structure, task-specific reasoning demands, and user needs. Based on this understanding, future research could focus on developing cognitively adaptive visualization systems that can dynamically adjust interface layouts according to users’ cognitive style, task complexity, and perceptual load (e.g., Steichen and Fu, 2019; Yelizarov and Gamayunov, 2014).

### 4.4 Design guidelines for comparative knowledge visualization

Findings from the present task context (static displays and propositional verification across three profiles) and the cognitive usability ratings indicate that no design strategy is universally superior for comparative knowledge visualizations. Rather, the effectiveness of a design strategy depends on its alignment with the primary epistemic goal and the task’s processing demands (cf. Meyer, 2010; Steiner and Albert, 2017a, 2017b). For example, users may need integrative comparison or source-specific inspection. Based on this goal-demand match, we derive four practical recommendations for structuring comparative displays that integrate and differentiate information from multiple sources.

First, superimposed layouts are advantageous when users must rapidly detect overlaps and gaps across profiles, particularly when the demands of comparison increase. In the present study, superimposition supported faster responses and higher accuracy under high task complexity across all complexity levels, which is consistent with the idea that perceptual alignment reduces the need for attentional shifting and mental integration during comparison (Gleicher et al., 2011; Windhager et al., 2020).

Second, juxtaposed layouts are preferable when users must attribute knowledge to specific contributors or inspect individual profiles with minimal interference. Juxtaposition preserves source separability, and it has been found to be clearer and more helpful for identifying individual knowledge. This suggests that its lower perceptual density can facilitate selective attention when source-specific inspection is the primary goal (Engelmann et al., 2009; Dehler Zufferey et al., 2010; Erkens and Bodemer, 2017, 2019).

Third, the absence of a significant difference in performance between concept maps and proposition lists in the present task suggests that representational effectiveness depends on task requirements rather than structural richness alone. For propositional verification tasks requiring the checking of discrete knowledge units, list-based formats may support efficient inspection through sequential, line-by-line access. Conversely, graph-based formats may be advantageous when the amount of conceptual content increases because node-link representations can reuse concepts across propositions, allowing for more compact scaling than sentence lists.

Fourth, when both epistemic goals-integrative overview and source attribution–are relevant within the same workflow, a plausible design response is an adaptive or hybrid interface that allows users to switch between layouts depending on the demands of the task or cognitive constraints (Otjacques and Feltz, 2005). For instance, users could start with a superimposed view to identify shared and unshared patterns at the group level, and then switch to a juxtaposed view to verify specific contributions with greater clarity and reduced interference.

### 4.5 Future research

This study examined how users performed and perceived different visual layouts when comparing distributed knowledge profiles. However, group knowledge visualizations are primarily used in collaborative contexts where reasoning is socially distributed, cognitively demanding, and highly dynamic. Therefore, future research could examine how the tested design dimensions–comparison strategy and knowledge format–support shared reasoning, joint attention, and effective coordination in real-world group settings (Janssen and Bodemer, 2013; Buder, 2017).

One avenue of research is to test the usability and epistemic effects of these design features in ecologically valid environments. Examples of these environments include interdisciplinary workshops, co-creation sessions, and instructional settings where participants explore and integrate conceptual knowledge collaboratively. Such settings would allow researchers to study cognitive outcomes and socio-epistemic phenomena, including awareness of knowledge asymmetries, negotiation of shared understanding, and attribution of informational responsibility (Bromme and Goldman, 2014; Engelmann et al., 2009).

To better understand the cognitive processes at play, future studies should use methods like eye tracking, interaction logging, and think-aloud protocols (Rayner, 1998; Holmqvist et al., 2011). These methods can reveal how users allocate attention, construct mental models, and resolve representational conflicts, especially in tasks requiring cross-profile integration or source-specific attribution. Cognitive modeling based on these methods could refine theoretical assumptions about perceptual alignment, working memory offloading, and integration effort (Sweller et al., 2019; Johnson-Laird, 1983; Cowan, 2010).

Another critical avenue of exploration is how individual user characteristics moderate the effectiveness of different layout configurations. Cognitive style (Mayer and Massa, 2003), metacognitive regulation (Flavell, 1979), and visualization literacy (Boy et al., 2014) may influence users’ ability to flexibly extract and integrate conceptual content, especially in complex situations. Adaptive visualization systems that respond to user profiles or reasoning phases could optimize layout structure dynamically and reduce unnecessary processing load (de Jong and van Joolingen, 1998; Bodemer and Scholvien, 2008).

In addition to person-based adaptivity, task-phase adaptivity presents another opportunity. Depending on whether users are exploring, comparing, or justifying knowledge, comparative visualizations may serve distinct cognitive functions. Dynamic systems that switch between layouts (e.g., from a superimposed map to a juxtaposed list) could better align with the evolving epistemic demands of distributed reasoning than static designs.

Together, these research directions highlight the importance of investigating comparative visualizations as epistemic tools whose effectiveness depends on their alignment with cognitive, social, and representational constraints, rather than merely as static information displays. Therefore, a cognitively informed design science of group visualizations must integrate insights from working memory theory, mental model theory, and visual attention research while grounding its designs in real-world contexts such as group reasoning and collaborative problem solving.

## 5 Conclusion

This study provides empirical evidence that the effectiveness of group knowledge visualizations in supporting their comparative function depends more on the chosen visual comparison strategy than on a conceptual-structural knowledge format. While concept maps and proposition lists yielded comparable performance in a propositional comparison task, the comparison strategy significantly influenced both accuracy and efficiency. Participants performed better with superimposed views, particularly as task complexity increased, suggesting that perceptual alignment is critical for managing cognitive load in knowledge comparison tasks.

The findings highlight a key insight for the design of collaborative visualization tools: effectiveness depends not merely on structural expressiveness but on the fit between visualization features and task demands. Superimposed layouts offer perceptual support for integration and synthesis, whereas juxtaposed layouts support separation and attribution. Designers could consider implementing hybrid or adaptive systems that allow users to shift between views depending on their current epistemic focus.

By integrating perspectives from cognitive load theory, visual cognition, and group awareness research, this study contributes to a better understanding of how knowledge representations and comparison strategies interact to support reasoning in distributed knowledge environments. Future research should examine how these findings scale to more complex, interactive, and socially dynamic settings, where visual reasoning, perspective-taking, and shared understanding must be negotiated in real time.

## Data Availability

The raw data supporting the conclusions of this article will be made available by the authors, without undue reservation.

## Footnotes

1
https://www.yworks.com

2
https://www.jspsych.org
