# Supplementary material for: Comparing distributed knowledge: the effects of visualization format and comparison strategy on task performance
Source: Front Psychol. 2026 Feb 10;17:1684634. doi: 10.3389/fpsyg.2026.1684634 (PMC12929453; doi:10.3389/fpsyg.2026.1684634)
Supplement: Supplementary file 1 [file Data_Sheet_1.pdf]

Supplementary Material S1:

We controlled for potential confounding effects of prior exposure to similar information formats on task performance in the visual comparison tasks. Participants rated their familiarity with statistical and network visualizations and tabular formats (see Section 2.3.3). Descriptively, a slightly higher percentage of participants in the Concept Map group reported more experience with statistical (37% vs. 31%) and network (11% vs. 4%) visualizations. Meanwhile, a slightly higher percentage of participants in the Proposition List group indicated more familiarity with tabular representations (21% vs. 15%). To determine whether these differences were statistically significant, we conducted three independent-samples t-tests. The results revealed no significant differences between the two groups for statistical data visualizations ($M = 3.33$ vs. 3.36), $t(125.25) = -0.12$, $p = .91$; network visualizations ($M = 2.84$ vs. 2.91), $t(120.86) = -0.34$, $p = .74$; or tabular formats ($M = 3.43$ vs. 3.60), $t(123.35) = -0.89$, $p = .38$. Therefore, we conclude that both groups were comparable in their prior experience with common information visualization formats, and differences in task performance are unlikely attributable to prior familiarity with these formats.
